# Extensive Phenotypic Variation among Allelic T-DNA Inserts in *Arabidopsis thaliana*

**DOI:** 10.1371/journal.pone.0044981

**Published:** 2012-09-13

**Authors:** Megan E. Valentine, Michael J. Wolyniak, Matthew T. Rutter

**Affiliations:** 1 Department of Biology, College of Charleston, Charleston, South Carolina, United States of America; 2 Department of Biology, Hampden-Sydney College, Hampden-Sydney, Virginia, United States of America; French National Centre for Scientific Research, Université Paris-Sud, France

## Abstract

T-DNA insertion mutants are a tool used widely in *Arabidopsis thaliana* to disrupt gene function. We phenotyped multiple homozygous T-DNA *A. thaliana* mutants at each of two loci (AT1G11060 and AT4G00210). We measured life history traits, including germination, size at reproduction and fruit production. Allelic T-DNA lines differed for most traits at AT1G11060 but not at AT4G00210. However, insertions in exons differed from other insertion positions in AT4G00210 but not in AT1G11060. We found evidence for additional insertions in approximately half of the lines, but found few phenotypic consequences. In general, our results suggest that a cautious interpretation of T-DNA phenotypes is warranted.

## Introduction

The production of mutants disabled at a single locus is the preeminent tool of reverse genetics. Examination of single knockout mutants has been useful in cellular and molecular biology, systems biology, genomics, and evolutionary biology [1]–[6]. A project led by the SALK institute is assembling a mutant collection with the goal of providing an insertion mutant for every identified gene in the *A. thaliana* genome [7]. Each line in their collection contains an insertion of *Agrobacterium* T-DNA in the *Arabidopsis* genome expressing a kanamycin resistance gene (*NPTII*) [8]. Currently, the SALK Homozygote T-DNA collection represents a set of confirmed homozygous mutant lines with at least one mutant for approximately 68% of *A. thaliana* loci [7], [9]. In most cases, the T-DNA insertion will likely cause a loss-of-function mutation [8], [9]. The T-DNA lines have proven especially useful at identifying loci contributing to phenotypes of interest (reviewed in [10]).

However, while the SALK T-DNA lines have been widely used to test gene function, there are reasons to be cautious in interpreting phenotypic data from a particular line, as has been noted by the developers of the lines [7], [11]. One reason for caution is due to the mechanism of the T-DNA insertion process [12], [13]. While the SALK institute determines the locus in which the insertion occurred by sequencing flanking regions [14], the insertion can be located in different genic regions: within an intron, exon or untranscribed regions such as promoters. The exact position of an insertion, and the type of sequence in which a particular insertion occurs can be found by entering the appropriate SALK line identifier at http://signal.salk.edu/cgi-bin/tdnaexpress. The “position effect” corresponding to the exact location of the insertion may have considerable phenotypic effect ([11], [15].

In addition, while the SALK T-DNA lines have been described as a “unimutant collection,” the presence of multiple insertions in a single line is a distinct possibility [7], [8]. T-DNA insertions can occur more than once during the insertion process. When the insertions were located, and subsequently screened for homozygosity, only a single insertion locus was identified. As many as 50% of lines may contain additional inserts at unknown loci [7], [15]. It is possible that a second locus may cause the phenotype of interest, or may alter the phenotypic effect of a knockout mutation.

Here we examine multiple homozygous alleles of T-DNA insertions from the SALK collection at two loci. We examine effects on life history phenotypes, including germination success, survival, time to flowering, and flower and fruit production. We found differences for these phenotypes among allelic lines at one locus, and evidence for position effects at the other, but detected few effects of additional insertions.

## Materials and Methods

### Plant Material

We obtained 13 SALK confirmed homozygous T-DNA insertion lines from two different loci and 1 control line lacking an insert (CS70000) (Table 1). Specifically we selected seven T-DNA lines associated with the AT1G11060 locus; and six lines with insertions in the AT4G00210 locus. The exact position of the insertion within the locus varied between the T-DNA lines, including insertions within exons, introns and untranscribed regions (UTRs). Insert positions were obtained from the SALK database (http://signal.salk.edu/cgi-bin/tdnaexpress).

**Table 1:** SALK T-DNA insertion lines used in this study, indicating the presence of multiple insertions and the insertion location within the locus.

AT1G11060			AT4G00210		
SALK Line ID	Insertion #	Insertion Location	SALK Line ID	Insertion #	Insertion Location
SALK_000755c	Multiple	Exon	SALK_056726c	Multiple	Intron
SALK_064701c	Single	UTR	SALK_067808c	Multiple	Intron
SALK_065229c	Multiple	Exon	SALK_076504c	Multiple	Exon
SALK_065856c	Single	Exon	SALK_082957c	Single	UTR
SALK_068953c	Single	Exon	SALK_126485c	Multiple	Exon
SALK_076791c	Single	Intron	SALK_128165c	Single	Exon
SALK_108385c	Single	Exon			

UTR  =  Untranscribed region.

### Phenotyping

Sixty seeds of each T-DNA line, and 60 seeds of the control Columbia CS70000 line were sown into 72-well flats and cold stratified for 10 days. Plants were removed from the cold and transferred to the greenhouse. Plants were scored for germination after one week in the greenhouse. After 3 weeks, 20 plants from each line were transplanted into 2.5″ square pots. We scored whether plants survived to flower and recorded days to bolting and flowering. We measured the rosette diameter when the plants initiated the bolting stem. After flowering was complete, the total number of rosette leaves was counted. Plants were then harvested and dried. We measured dried biomass, total branch number, total flowers produced, total aborted fruit, and total complete fruit.

### Analysis of T-DNA Insert Copy Number using Quantitative Dual-target PCR

To identify and analyze lines that possess multiple T-DNA inserts, a quantitative dual-target PCR (QD-PCR) technique was used based on procedures developed by Kihara *et al.* [16]. Briefly, two sets of primers were simultaneously used in a PCR reaction with *Arabidopsis* genomic DNA. One primer set corresponded to a known single-copy reference gene in the *Arabidopsis* genome encoding either phosphosynthetic electron transfer c (PetC) or 4-hydroxyphenylpyruvate dioxygenase (4HPPD) and produce a ∼500 basepair PCR product. The other primer set corresponded to the T-DNA insertion cassette and produced a 624 basepair product. The presence of multiple T-DNA inserts in a given line was determined by analyzing the relative intensities of the resultant PCR products on an agarose gel. Previous work confirmed the accuracy of this approach with qPCR and found it to be reliable and reproducible [16].

### Statistical Analyses

Tests for differences among lines were performed within each locus. We tested for differences between single and multiple insertions, and between insertion location categories (exons vs. introns or untranscribed regions). Response variables included germination, survival, rosette diameter at bolting, days to bolting, branch number, flower number, fruit number, percent fruits aborted, and dry biomass. Binary response variables, such as germination and survival, were analyzed within the LOGISTIC procedure of SAS (v. 9.2). Most other variables had a normal distribution and were analyzed with ANOVA within the GLM procedure. Exceptions included branch number, aborted fruit number and total flower number which were analyzed with a Poisson response distribution and log link function within the GENMOD procedure. All analyses used fully fixed models. P-values were determined from orthogonal contrasts between categories within the overall model.

## Results

**Figure 1:**
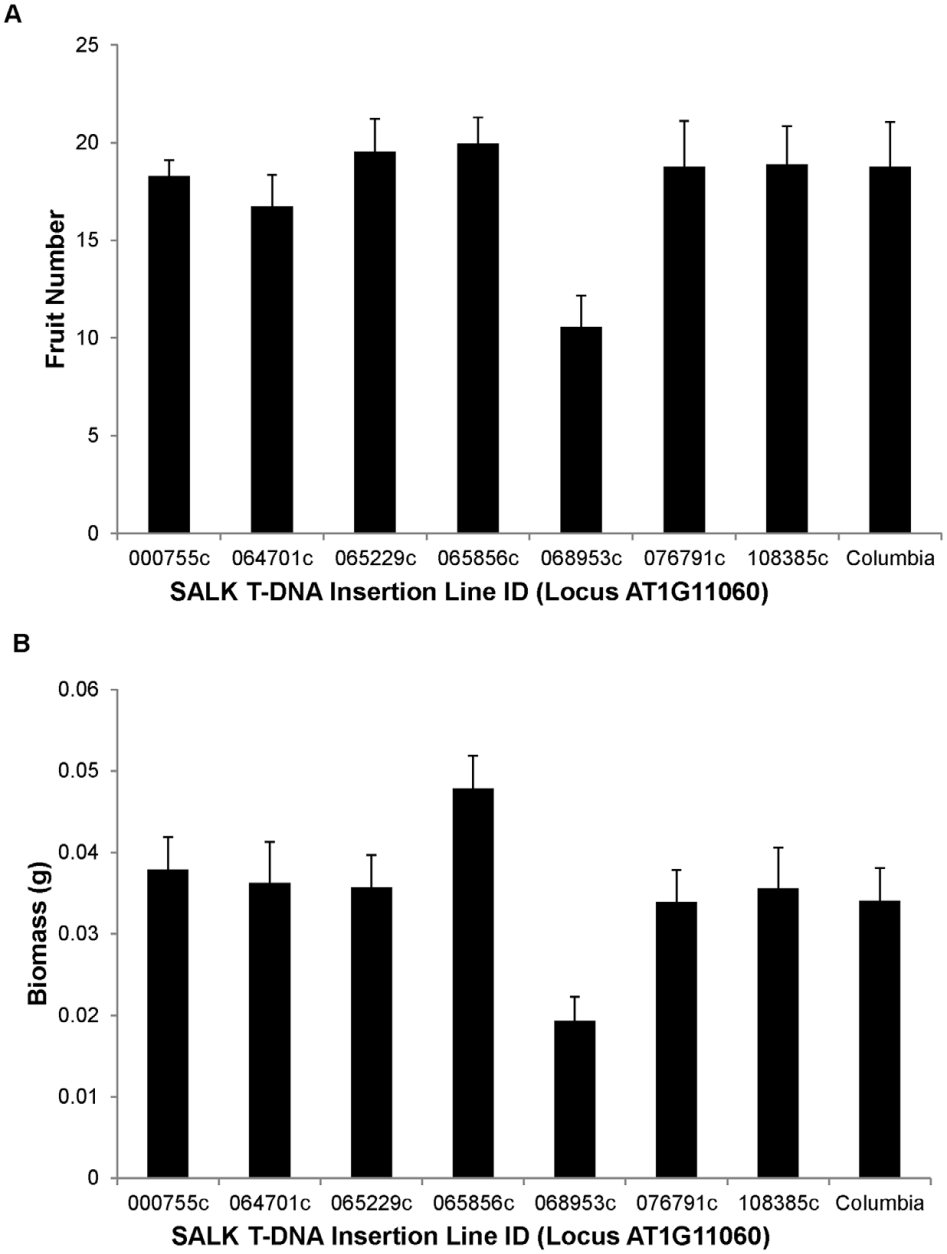
Variation among T-DNA insertion alleles. The average phenotype for T-NA insertion lines at locus AT 1G11060 and wild-type Columbia for A) fruit number and B) biomass. Error bars indicate standard errors.

Within locus AT1G11060, homozygous T-DNA insertion lines varied significantly for most traits (see Figure 1 for examples). Lines varied for germination (P = 0.0003), biomass (P = 0.0007), measures of rosette diameter at two time points (P<0.0001 for both time points), total number of leaves (P<0.0001), total fruit production (P = 0.0032), total flower production (P = 0.011), number of aborted fruits (P<0.0001) (see Table S1 for all trait means and standard errors in each line). Within locus AT4G00210, there was far less variation between the insertion lines. Germination was the only character measured that varied significantly between lines at this locus (P<0.0001). Branch number and the days to bolting did not vary significantly between lines for either locus.

**Figure 2:**
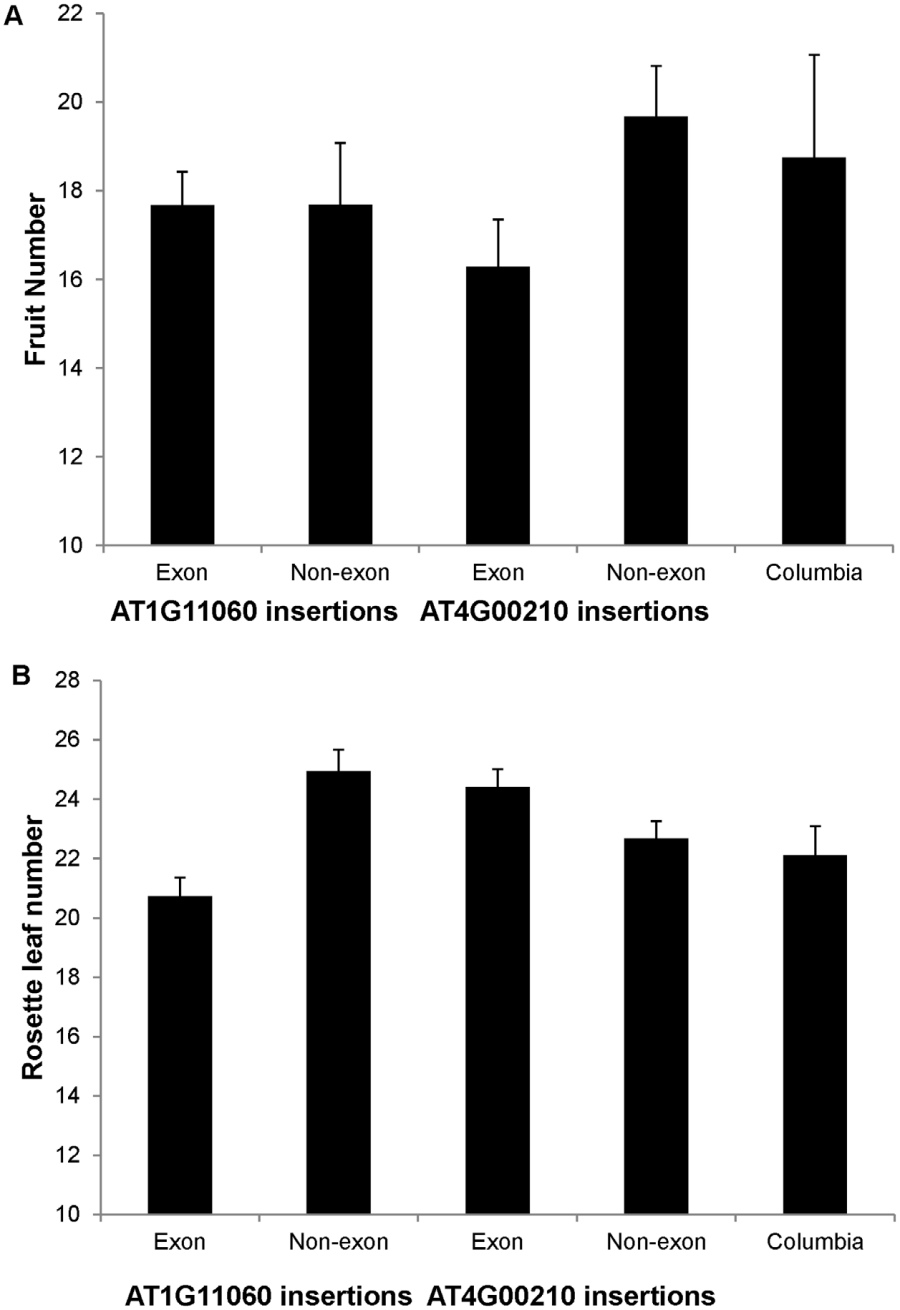
Position effects of T-DNA insertion. Phenotypes of T-DNA lines with exonic insertions and non-exonic insertions for A) fruit number and B) rosette leaf number. Wild-type Columbia phenotypes are also shown. Error bars indicate standard errors.

When lines within each locus were grouped by the position of the insertion (i.e. exon insertions vs. other types of insertions), the results varied between the loci (Figure 2). For AT1G11060, plants with insertions in exons had fewer leaves (P<0.0001) and higher germination rates (P = 0.0131) than plants with insertions in other locations. For AT4G00210, plants with insertion in exons had more leaves (P = 0.0335), but lower fruit number (P = 0.0279) and germination rates (P<0.0001). Other traits did not differ significantly by insertion position for either locus.

As expected, about half the loci had more than one insertion (Table 1). However, there were only two detectable effects of an additional insertion on phenotype. Within locus AT4G00210, plants with single insertions germinated at a lower rate than plants with multiple insertions (P<0.0001). Within locus AT1G11060, plants with multiple insertions had more flowers (P = 0.0016).

## Discussion

We found that, within a locus, alleles of the confirmed homozygous SALK T-DNA lines vary significantly in several phenotypes. Of the two loci surveyed, one (AT1G11060) appeared to have much greater variability across lines. AT1G11060 is a Wings-apart-like (Wapl) protein involved in regulation of heterochromatin. The function of Wapl proteins has been investigated in animals and yeast [17], where they have been implicated in the dissociation of the binding between the protein cohesin and chromatin. In vertebrates, if Wapl is depleted cohesin does not dissociate from the chromatin and dissociation of sister chromatids is delayed [18]. The function of Wapl in plants has not yet been explored. AT4G00210 contains a lateral organ bounding (LOB) domain, part of a gene family unique to plants with several dozen members in *A. thaliana* [19]. Although AT4G00210 (also known as *LBD31* for Lob Binding Domain 31) has not been functionally characterized, other members of the family are known to be involved in leaf, root and flower formation and development and in responses to auxin, cytokinins and gibberellins [20].

Part of the variation among lines within a locus could be attributed to differences in the type of insertion. In general, our results were consistent with other evidence that insertions within exons are more likely to have phenotype than insertions in other regions [11]. However, even within the class of exon insertions, phenotypes were still highly variable depending on the exact insertion position, as has been noted for other Arabidopsis T-DNA mutants [11]. Although even confirmed lines are frequently expected to harbor additional unknown mutations, we detected few effects of additional T-DNAs on phenotypes. Since only about 40% of the A. thaliana genome is coding sequence [21], it is likely that a significant number of additional unidentified insertions would not be in a second gene. The additional insertions are thus would be less likely to affect phenotypes, which would be consistent with our results. However, because our results are based on an examination of only two loci, it is difficult to determine the generality of our findings- as the extent of positional effects or of multiple insertions might differ substantially at other loci or in other lines.

Our study provides support for a very cautious interpretation of phenotypic effects ascribed to particular T-DNA insertions. While these insertions have been a phenomenal resource for the plant biology community, they are best viewed as a first step in linking genotypic change to phenotypic consequence. For example, using the SALK line SALK_064701c alone, it would appear that AT1G11060 does not affect fruit number (in this line, fruit production was identical to wild-type Columbia). A lack of phenotype would be consistent with other findings that insertions in untranscribed promoter regions frequently do not alter transcription or protein formation [11]. However, if SALK lines SALK_65856c or SALK_68953c had been used in an experiment a researcher might conclude that AT1G11060 is critical to fruit production. However, the conclusions drawn still would have depended on line choice–fruit production was higher in than wild type in SALK_65856c but lower in SALK_68953c. Both of these mutations were also insertions in exons. We echo the recommendation of others [7], [10] that verification of phenotypic effects of T-DNA insertions with alternative alleles at the same locus is a critical component to reverse genetics. Further investigation of a larger group of loci with multiple T-DNA alleles would clarify the extent to which such differences contribute to observed phenotypes.

## Supporting Information

Table S1 Mean values of each measured trait in each line. Standard errors are in parentheses.
(XLSX)
